# Tuberculosis and HIV Co-infection, California, USA, 1993–2008

**DOI:** 10.3201/eid1903.121521

**Published:** 2013-03

**Authors:** John Z. Metcalfe, Travis C. Porco, Janice Westenhouse, Mark Damesyn, Matt Facer, Julia Hill, Qiang Xia, James P. Watt, Philip C. Hopewell, Jennifer Flood

**Affiliations:** Author affiliations: University of California, San Francisco, California, USA (J.Z. Metcalfe, T.C. Porco, P.C. Hopewell); California Department of Public Health, Richmond, California, USA (J. Westenhouse, J. Hill, J.P. Watt, J. Flood);; California Department of Public Health, Sacramento, California, USA (M. Damesyn, M. Facer);; RTI International, Research Triangle Park, North Carolina, USA (Q. Xia)

**Keywords:** Tuberculosis, HIV, TB–HIV co-morbidity, HAART, California, Mycobacterium tuberculosis and other mycobacterial diseases, United States, co-infection, USA, highly active antiretroviral therapy

## Abstract

To understand the epidemiology of tuberculosis (TB) and HIV co-infection in California, we cross-matched incident TB cases reported to state surveillance systems during 1993–2008 with cases in the state HIV/AIDS registry. Of 57,527 TB case-patients, 3,904 (7%) had known HIV infection. TB rates for persons with HIV declined from 437 to 126 cases/100,000 persons during 1993–2008; rates were highest for Hispanics (225/100,000) and Blacks (148/100,000). Patients co-infected with TB–HIV during 2001–2008 were significantly more likely than those infected before highly active antiretroviral therapy became available to be foreign born, Hispanic, or Asian/Pacific Islander and to have pyrazinamide-monoresistant TB. Death rates decreased after highly active antiretroviral therapy became available but remained twice that for TB patients without HIV infection and higher for women. In California, HIV-associated TB has concentrated among persons from low and middle income countries who often acquire HIV infection in the peri-immigration period.

The modern resurgence of tuberculosis (TB) in conjunction with the HIV pandemic remains a major public health dilemma. In 2011, nine percent of all newly reported TB cases in the United Sates for which HIV status was known (*1*) and 13% (1.1 million cases) of cases reported worldwide (*2*) were associated with HIV co-infection. Despite compelling declines in TB incidence and associated deaths with use of highly active antiretroviral therapy (HAART), TB remains the leading cause of death among persons with HIV/AIDS (*3*,*4*).

California, the most populous state in the United States (38 million persons [12% of the US population]), reports the highest annual number of persons with TB (22.1% of total) and the second highest number of HIV-infected persons (103,073 [12.4%] cases) (*5*). Yet, because of prior restrictions on HIV reporting and limited systematic linking of state TB and HIV surveillance systems, California has not been included in key national surveillance reports of HIV incidence (*6*) or death in persons with TB–HIV (*7*).

Effective control of TB–HIV requires an understanding of the changing epidemiology of these diseases. To provide information for disease-reduction efforts and to improve survival among persons with TB–HIV, we retrospectively reviewed all incident TB–HIV cases in a 16-year period in California during which dynamic changes occurred in the HIV epidemic as a consequence of the introduction of HAART.

## Methods

### Study Population

We analyzed all TB cases reported to the California TB registry during January 1, 1993–December 31, 2008. California state law requires reporting of all verified cases of TB and HIV/AIDS (California Health and Safety Code Title 17§2505, and Section 121022 [2006]) to their respective programs. TB–HIV patients were identified through a statewide registry match with the California Office of AIDS by using Registry Plus Link Plus software (Technical Appendix). Annual state HIV prevalence was estimated through nonparametric back-calculation based on racial/ethnic group–specific counts of reported AIDS cases and reported AIDS-related deaths during 1981–2008 (Technical Appendix Table 1) (*8*). Demographic, behavioral, and clinical information, including deaths, was abstracted from state surveillance forms (Report of a Verified Case of Tuberculosis and Adult HIV/AIDS Confidential Case Report). Surveillance data for both diseases have demonstrated high validity (*9*,*10*).

**Table 1:** Demographic characteristics of TB patients with and without known HIV co-infection, California, 1993–2008*

Characteristic	TB without known HIV			TB with HIV			
	1993–1995, n = 13,297	1996–2000, n = 17,768	2001–2008, n = 22,558	1993–1995, n = 1,343	1996–2000, n = 1,307	2001–2008, n = 1,254	p value†
Median age, y (IQR)	42 (27–62)	45 (29–64)‡	47 (30–64)‡	37 (31–44)	38 (33–45)‡	40 (34–48)‡	0.8
Female sex	5,343 (40)	7,249 (41)	9,294 (41)	147 (11)	177 (14)	217 (17)	<0.001
	Referent	1.0 (0.99–1.04)	1.03 (1.00–1.05)	Referent	1.2 (1.0–1.5)	1.6 (1.3–1.9)	
Race/Ethnicity							
Asian/Pacific Islander	4,972 (37)	7,429 (42)	10,091 (45)	52 (4)	59 (5)	105 (8)	0.002
	Referent	1.12 (1.09–1.15)	1.19 (1.16–1.22)	Referent	1.2 (0.8–1.7)	2.2 (1.6–3.0)	
Hispanic	4,955 (38)	6,306 (35)	8,519 (38)	512 (38)	619 (47)	714 (57)	<0.001
	Referent	1.0 (0.9–1.0)	1.01 (0.98–1.04)	Referent	1.2 (1.1–1.4)	1.5 (1.4–1.6)	
Black, non-Hispanic	1,483 (11)	1,674 (10)	1,628 (7)	440 (33)	369 (28)	265 (21)	0.18
	Referent	0.9 (0.8–0.9)	0.7 (0.6–0.7)	Referent	0.9 (0.8–1.0)	0.6 (0.6–0.7)	
White, non-Hispanic	1,776 (13)	2,197 (12)	2,174 (10)	327 (24)	252 (19)	161 (13)	<0.001
	Referent	0.9 (0.9–1.0)	0.7 (0.7–0.8)	Referent	0.8 (0.7–0.9)	0.5 (0.4–0.6)	
Foreign born	8,951 (67)	12,635 (71)	17,296 (77)	488 (36)	597 (46)	784 (63)	<0.001
	Referent	1.1 (1.0–1.1)	1.1 (1.1–1.2)	Referent	1.3 (1.2–1.4)	1.7 (1.6–1.9)	
Country/region of origin							
Mexico	2,909 (32)	3,826 (30)	5,274 (30)	295 (60)	385 (64)	502 (64)	0.07
	Referent	0.9 (0.9–1.0)	0.9 (0.9–1.0)	Referent	1.1 (1.0–1.2)	1.1 (1.0–1.2)	
Central America	521 (6)	611 (5)	937 (5)	77 (16)	84 (14)	93 (12)	0.05
	Referent	0.8 (0.7–0.9)	0.9 (0.8–1.0)	Referent	0.9 (0.7–1.2)	0.8 (0.6–1.0)	
Philippines	1,745 (19)	2,669 (21)	3,580 (21)	27 (6)	28 (5)	43 (5)	0.44
	Referent	1.1 (1.0–1.1)	1.1 (1.0–1.1)	Referent	0.8 (0.5–1.4)	1.0 (0.6–1.6)	
People’s Republic of China	504 (6)	774 (6)	1,108 (6)	3 (0.6)	2 (0.3)	2 (0.3)	0.26
	Referent	1.1 (1.0–1.2)	1.1 (1.0–1.3)	Referent	0.5 (0.1–3.3)	0.4 (0.1–2.5)	
Mainland Southeast Asia	1,770 (20)	2,391 (19)	2,910 (17)	10 (2)	21 (4)	39 (5)	<0.001
	Referent	1.0 (0.9–1.0)	0.9 (0.8–0.9)	Referent	1.7 (0.8–3.6)	2.4 (1.2–4.8)	
Sub-Saharan Africa	92 (1)	181 (1)	331 (2)	7 (1)	25 (4)	52 (7)	0.06
	Referent	1.4 (1.1–1.8)	1.9 (1.5–2.3)	Referent	2.9 (1.3–6.7)	4.6 (2.1–10.1)	
Median time from US entry to TB diagnosis, y (IQR)	5.3 (1–13.6)	8.2 (2.2–17.1)‡	10 (2.2–20.8)‡	9.8 (5–15.4)	10.5 (4.5–18.5)	10.9 (3–20.7)	0.001
HIV risk group§							
MSM	NA	NA	NA	634 (47)	578 (44)	518 (41)	NA
	NA	NA	NA	Referent	0.9 (0.9–1.0)	0.9 (0.8–1.0)	
IDU	NA	NA	NA	289 (22)	276 (21)	186 (15)	NA
	NA	NA	NA	Referent	1.0 (0.8–1.1)	0.7 (0.6–0.8)	
MSM and IDU	NA	NA	NA	218 (16)	137 (10)	105 (8)	NA
	NA	NA	NA	Referent	0.7 (0.5–0.8)	0.5 (0.4–0.6)	
Heterosexual contact	NA	NA	NA	84 (6)	112 (9)	206 (16)	NA
	NA	NA	NA	Referent	1.4 (1.0–1.8)	2.6 (2.1–3.3)	
Unknown	NA	NA	NA	92 (7)	188 (14)	223 (18)	NA
	NA	NA	NA	Referent	2.1 (1.7–2.7)	2.6 (2.1–3.3)	
Homeless	930 (7)	1,059 (6)	1,270 (6)	244 (18)	233 (18)	239 (19)	0.01
	Referent	0.9 (0.8–0.9)	0.8 (0.7–0.9)	Referent	1.0 (0.8–1.2)	1.1 (0.9–1.2)	
Excess alcohol use	1,270 (10)	1,897 (11)	2,071 (9)	255 (19)	298 (23)	255 (20)	0.8
	Referent	1.1 (1.0–1.2)	1.0 (0.9–1.0)	Referent	1.2 (1.0–1.4)	1.1 (0.9–1.3)	

*TB diagnoses were grouped into 3 periods: pre-HAART (1993–1995), early HAART (1996–2000), and late HAART (2001–2008). All values are no. (%)/prevalence ratio (95% CI) unless otherwise indicated. The denominator for each characteristic excludes missing or unknown values. TB, tuberculosis; IQR, interquartile range; MSM, men who have sex with men; NA, not applicable; IDU, injection drug use. †Rates of change in prevalence of binary covariates among patients with TB vs. patients with TB–HIV were compared by using logistic regression with robust SEs; similarly, rates of change in continuous covariates among patients with TB vs. patients with TB–HIV were compared by using linear regression. For difference in annual percentage change (TB vs. TB–HIV) ‡p<0.001 difference in medians relative to early time period. §Other exposure categories are hemophilia/coagulation disorder; receipt of blood, components, or tissue; and perinatal transmission. These accounted for <2% of suspected risk factors for HIV acquisition during each time period and are not included in the table.

### Definitions

HIV cases were classified in accordance with current Centers for Disease Control and Prevention (CDC) surveillance case definitions (*11*). For this analysis, patients not identified in the California HIV/AIDS registry were considered HIV-negative. TB diagnoses were grouped into 3 periods on the basis of HAART availability and approximately equal distribution of TB–HIV cases: pre-HAART (1993–1995), early HAART (1996–2000), and late HAART (2001–2008). Late diagnosis of HIV infection was defined as an AIDS diagnosis made <12 months after an initial diagnosis of HIV infection. Advanced immunosuppression was defined as a CD4+ T-lymphocyte count <50 cells/mm^3^; valid CD4+ T-lymphocyte counts were those collected within 6 months of TB diagnosis. Drug susceptibility testing was performed at local laboratories or at the California Microbial Diseases Laboratory (Richmond, CA, USA) by using the BACTEC 460TB System (Becton Dickinson Diagnostic Instruments, Sparks, MD, USA), BACTEC MGIT 960 MycoBacterial Detection System (Becton Dickinson), or the agar proportion method.

### Statistical Analysis

Stratum-specific TB incidence per 100,000 population was calculated by dividing the number of incident cases by total (*12*) and HIV-infected population denominators. Clinical trends and demographic characteristics were described in 2 ways. First, we calculated prevalence ratios (PRs) and 95% CIs for comparison of characteristics associated with TB and TB–HIV cases (*13*). Second, we compared rates of annual percentage change (1993–2008) in prevalence of binary covariates by using logistic regression with robust SEs. This model included main effects for year (as a categorical variable) and HIV infection status, as well as the interaction between them.

Multivariate associations with death among TB–HIV patients were examined by using a generalized linear model with a log link and robust SEs to generate relative risk (RR) estimates (*14*); the model was a priori specified to include time period, age, sex, race/ethnicity, foreign birth, HIV risk factor, CD4+ T-lymphocyte count, sputum smear positivity, and interval between the diagnoses of HIV infection and TB. Death at diagnosis or during treatment was calculated from patients for whom outcome was known (3,754/3,904 [96.2%]); clinical outcomes during 2007 were excluded because of incomplete reporting. Multiple imputation was used to impute missing values for CD4+ T-lymphocyte count and viral load (*15*). The results obtained after multiple imputation were compared with those from an unimputed complete-case analysis (Technical Appendix Table 2).

**Table 2:** Clinical characteristics of TB patients with and without known HIV co-infection, California, USA, 1993–2008*

Characteristic	TB without known HIV				TB with HIV			p value†
	1993–1995, n = 13,297	1996–2000, n = 17,768	2001–2008, n = 22,558		1993–1995, n = 1,343	1996–2000, n = 1,307	2001–2008, n = 1,254	
Extrapulmonary TB‡	2,343 (18)	3,351 (19)	4,621 (21)		222 (17)	230 (18)	213 (17)	0.11
	Referent	1.07 (1.02–1.12)	1.16 (1.11–1.22)		Referent	1.06 (0.9–1.26)	1.03 (0.87–1.22)	
Pulmonary TB								
AFB smear-positive	4,200 (45)	6,002 (47)	8,338 (50)		577 (55)	531 (52)	546 (53)	<0.01
	Referent	1.04 (1.01–1.07)	1.10 (1.07–1.13)		Referent	0.94 (0.87–1.02)	0.97 (0.89–1.05)	
AFB smear-negative, culture positive	3,180 (34)	4,301 (34)	5,093 (31)		347 (33)	339 (33)	325 (32)	0.37
	Referent	0.98 (0.95–1.02)	0.90 (0.87–0.93)		Referent	1.00 (0.89–1.13)	0.96 (0.85–1.08)	
Culture-negative§	2,216 (21)	2,805 (20)	3,477(20)		110 (10)	155 (15)	167 (16)	0.001
	Referent	0.94 (0.90–0.99)	0.92 (0.88–0.97)		Referent	1.16 (0.94–1.44)	1.29 (1.05–1.59)	
Cavitary disease	2,255 (21)	2, 981 (21)	4,128 (23)		117 (11)	79 (7)	97 (9)	0.21
	Referent	1.00 (0.95–1.05)	1.12 (1.07–1.17)		Referent	0.70 (0.53–0.91)	0.88 (0.68–1.14)	
Median time to culture conversion, mo (IQR)	1.9 (1.0–3.4)	1.8 (0.9–2.9)	1.5 (0.8–2.4)		2.0 (1.1–4.2)	1.5 (0.8–2.8)	1.2 (0.7–2.3)	0.19
Median CD4 count, cells/mm^3^ (IQR)¶	NA	NA	NA		114 (60–179)	103 (52–162)	100 (55–150)**	NA
Viral load¶	NA	NA	NA		50,282 (21,203–97,644)	68,501 (28,054–119,655) **	83,402 (28,994–177,698) **	NA
Drug resistance#								
INH resistant**	832 (8)	1,268 (8)	1,541 (8)		55 (5)	65 (5)	72 (6)	0.10
	Referent	1.07 (0.98–1.16)	1.02 (0.94–1.11)		Referent	1.15 (0.81–1.63)	1.34 (0.95–1.89)	
PZA nonoresistance	138 (1)	322 (2)	636 (3)		24 (2)	68 (6)	93 (8)	<0.05
	Referent	1.63 (1.34–2.00)	2.53 (2.11–3.04)		Referent	2.74 (1.73–4.33)	3.94 (2.53–6.13)	
MDR	148 (1)	186 (1)	257 (1)		9 (1)	7 (1)	10 (1)	0.90
	Referent	0.88 (0.71–1.09)	0.96 (0.78–1.17)		Referent	0.76 (0.28–2.03)	1.14 (0.46–2.79)	
Deceased at diagnosis	319 (2.4)	406 (2.3)	359 (1.6)		50 (3.7)	40 (3.1)	26 (2.1)	0.50
	Referent	0.95 (0.82–1.10)	0.66 (0.57–0.77)		Referent	0.82 (0.55–1.24)	0.56 (0.35–0.89)	
Final treatment outcome††								
Completed treatment	10,598 (83)	14,807 (87)	18,048 (87)		713 (56)	937 (75)	901 (78) 1.39 (1.31–1.47)	<0.001
	Referent	1.04 (1.03–1.05)	1.05 (1.04–1.06)		Referent	1.34 (1.27–1.42)		
Defaulted	519 (4)	334 (2)	480 (2)		60 (5)	37 (3)	38 (3)	0.61
	Referent	0.48 (0.42–0.55)	0.57 (0.50–0.64)		Referent	0.63 (0.42–0.94)	0.70 (0.47–1.04)	
Transferred	780 (6)	775 (5)	842 (4)		121 (10)	79 (6)	59 (5)	0.19
	Referent	0.74 (0.67–0.82)	0.66 (0.60–0.73)		Referent	0.67 (0.51–0.88)	0.54 (0.40–0.72)	
Died	864 (7)	1,161 (7)	1,394 (7)		375 (30)	190 (15)	158 (14)	<0.001
	Referent	1.00 (0.92–1.09)	0.99 (0.91–1.08)		Referent	0.52 (0.44–0.61)	0.46 (0.39–0.55)	

*TB diagnoses were grouped into 3 periods: before the availability of highly active antiretroviral therapy (HAART) (1993–1995), early HAART (1996–2000), and late HAART (2001–2008). All values are no. (%)/prevalence ratio (95% CI). The denominator for each characteristic excludes missing or unknown values. TB, tuberculosis; AFB, acid-fast bacilli; IQR, interquartile range; NA, not applicable; INH, isoniazid; PZA, pyrazinamide; MDR, multidrug-resistant. †p value for difference (TB vs. TB–HIV). Rates of change in prevalence of binary covariates among patients with TB versus patients with TB–HIV were compared by using logistic regression with robust SEs; similarly, rates of change in continuous covariates among patients with TB versus patients with TB–HIV were compared by using linear regression. ‡Without evidence of pulmonary TB. §Comprising pulmonary and extrapulmonary TB. ¶CD4 count and viral load were imputed (online Technical Appendix, wwwnc.cdc.gov/EID/articles/12-1521-Techapp1.pdf). #Denominator includes only culture-positive cases. **Excluding multidrug resistance (defined as laboratory-confirmed resistance to INH and rifampin) and extensive drug resistance (defined as laboratory-confirmed resistance to all of the following, at minimum: INH, rifampin, any fluoroquinolone, and any second-line injectable agent) ††Limited to TB patients who were alive at diagnosis; treatment outcomes for 2007 were incomplete and were excluded.

Proportions were compared by using χ^2^ tests, and continuous variables were compared by using the Wilcoxon rank-sum test. All p values were 2-sided with α = 0.05 as the significance level. Data were analyzed by using Stata 12.1 (Stata Corporation, College Station, TX, USA) and R version 2.13.2 (R Foundations for Statistical Computing, Vienna, Austria).

## Results

**Figure 1:**
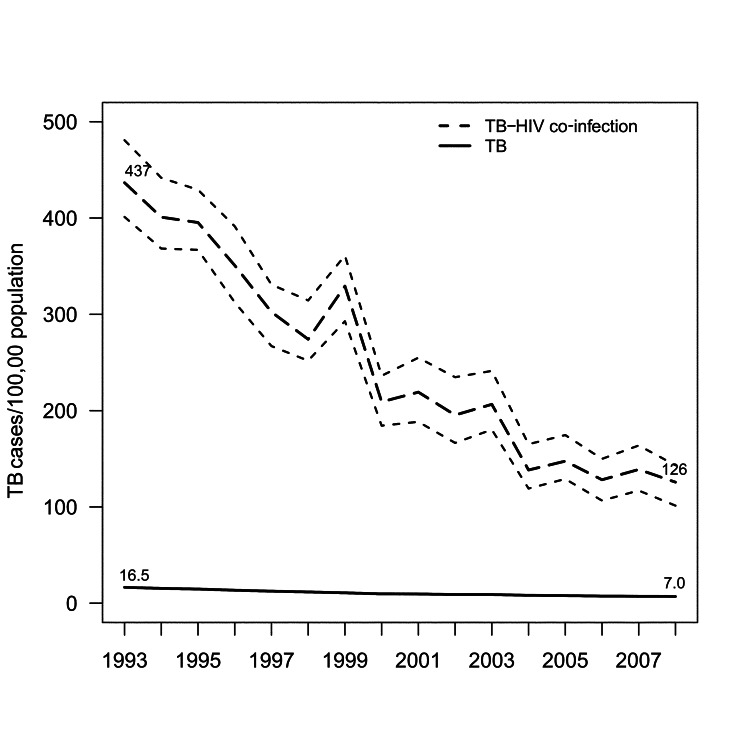
Rates of tuberculosis (TB) and TB–HIV, California, USA, 1993–2008. Area between dashed lines represents 95% bootstrap percentile CIs for TB–HIV rates. Annual state HIV prevalence was estimated through nonparametric back-calculation based on racial/ethnic group–specific counts of reported AIDS cases and reported AIDS-related deaths during 1981–2008 (online Technical Appendix, wwwnc.cdc.gov/EID/article/19/3/12-1521-Techapp1.pdf).

**Figure 2:**
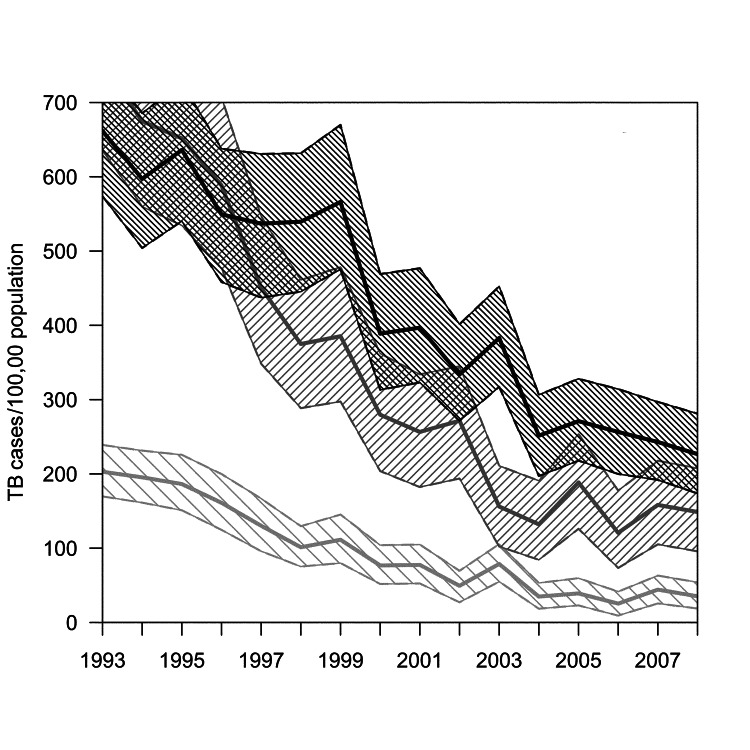
Rates of tuberculosis (TB) for persons with HIV/AIDS, California, USA, 1993–2008. Shaded areas represent 95% bootstrap percentile CIs, by race. TB–HIV rates for Asians/Pacific Islanders could not be calculated because of small numbers of cases during some years. Annual state HIV prevalence was estimated through nonparametric back-calculation on the basis of racial/ethnic group–specific counts of reported AIDS cases and reported AIDS-related deaths during 1981–2008 (online Technical Appendix, wwwnc.cdc.gov/EID/article/19/3/12-1521-Techapp1.pdf). Light gray, Whites; medium gray, Blacks; dark gray, Hispanics.

During 1993–2008, a total of 57,527 TB cases occurred in California, 3,904 (6.8%) of which were identified in a registry match with the California Office of AIDS. Overall, the proportion of TB–HIV cases decreased from 1,343 (9.2%) of 14,640 in the pre-HAART era to 1,254 (5.3%) of 23,812 in late HAART era. TB incidence among patients with HIV/AIDS declined from 437 cases per 100,000 persons to 126 per 100,000 (71% decrease); TB incidence rates for persons without known HIV co-infection declined from 16.5 cases per 100,000 persons to 7.0 per 100,000 (58% decrease) (Figure 1). Throughout the study period, incidence declined markedly in all strata of race/ethnicity but remained highest among Hispanics (225/100,000) and Blacks (148/100,000) with HIV/AIDS (Figure 2).

### Demographic Trends

The median age of patients with known TB–HIV increased throughout the study period (Table 1); the number of persons >50 years of age with TB–HIV increased from 12% in the pre-HAART era to 21% in the late HAART era, out of proportion to the increase (from 40% to 46%) for TB cases alone (p<0.001 for difference in slopes). Likewise, female patients with TB–HIV increased from 11% to 17%, out of proportion to the increase (from 40% to 41%) for TB cases alone (p<0.001 for difference in slopes).

#### Foreign-born Persons

The proportion of TB–HIV patients who were foreign born increased from 37% in the pre-HAART era to 63% in the late HAART era, a greater increase than for TB patients without known HIV co-infection (from 67% to 77%; p<0.001 for difference in slopes). Seventy-six percent of foreign-born patients with TB–HIV (and 37% of all patients with TB–HIV) immigrated from Mexico or Central America. Among persons with HIV infection, TB was diagnosed a median of 11 years (interquartile range [IQR] 4–19 years) after entry into the United States, significantly longer than for persons without known HIV infection (8 years [IQR 2–18 years], p<0.001 by Wilcoxon rank-sum test). In contrast, TB–HIV patients were consistently younger (25 years [IQR 19–33 years]) than TB patients (31 years [IQR 20–49 years]; p<0.001 by Wilcoxon rank-sum test) at time of US entry. Immigrants from Mexico or Central America who had TB–HIV were younger (23 years [IQR 17–35 years]) than those from Southeast Asia or sub-Saharan Africa (36 years [IQR 24–55 years]; p<0.001 by Wilcoxon rank-sum test). In HIV-infected persons from sub-Saharan Africa, active TB developed sooner after immigration (2.6 years [IQR 0.2–5.7 years]) than in persons from all other regions (10.8 years [IQR 4.5–19.3 years]; p<0.001 by Wilcoxon rank-sum test). Compared with the pre-HAART era, patients with TB–HIV in the late HAART era were more likely to originate from Southeast Asia (PR 2.4; 95% CI 1.2–4.8) or sub-Saharan Africa (PR 4.6; 95% CI 2.1–10.1).

#### Race/Ethnicity

Hispanics accounted for 57% of all TB–HIV cases in the late HAART era (a 20% increase from the pre-HAART era); in contrast, the proportion of TB patients without known HIV co-infection who were Hispanic (38%) did not change. Asian/Pacific Islanders also represented an increasing proportion of TB–HIV patients in the late from the early HAART eras (8% vs. 4%; PR 2.2 [95% CI 1.6–3.0]). Among US-born persons, TB–HIV declined among Blacks (pre-HAART vs. late HAART; 31% vs. 17%) and Whites (23% vs. 12%) but not Hispanics (8% vs. 8%; p<0.001 for difference in slopes).

#### HIV Risk Group

From the pre-HAART era to the late HAART era among TB–HIV patients, the HIV transmission risk factors of men who have sex with men (MSM) (47% vs. 41%), injection drug use (IDU) (22% vs. 15%), and MSM/IDU (16% vs. 8%) decreased, whereas presumed heterosexual transmission increased (6% vs. 16%; p<0.01 by χ^2^). MSM was the most commonly reported HIV risk factor for all racial/ethnic groups. Presumed heterosexual transmission increased over time and was more common for TB–HIV cases diagnosed in the late HAART era among Hispanics (12%; 95% CI 10%–13%) and Blacks (11%; 95% CI 9%–13%) than among Whites (4%; 95% CI 3%–5%). Similarly, unknown or unreported HIV transmission risk factors were more common among Hispanics (16%; 95% CI 15%–18%) and Blacks (10%; 95% CI 9%–12%) than among Whites (5%; 95% CI 4%–7%).

### Clinical Trends

#### HIV Characteristics

Overall, AIDS developed within 12 months after HIV diagnosis in 72% of patients, a percentage that did not substantially change throughout the study. Median CD4+ T-lymphocyte count was 114 cells/mm^3^ (IQR 60–179 cells/mm^3^) during the pre-HAART era and 100 cells/mm^3^ (IQR 55–150; p<0.01 by Wilcoxon rank-sum test) during the late HAART era (Table 2). Approximately 20% of patients had advanced immunosuppression (CD4+ T-lymphocyte count <50 cells/mm^3^) during both the pre-HAART and late HAART eras.

#### TB Characteristics

Patients with TB–HIV were more likely to be sputum smear positive (PR 1.11; 95% CI 1.05–1.17) and less likely to have culture-negative pulmonary TB (PR 0.58; 95% CI 0.49–0.68) than patients without known HIV co-infection during the pre-HAART era. These differences diminished in the late HAART era (PR 1.01; 95% CI 0.96–1.07 and PR 0.81; 95% CI 0.70–0.93, respectively).

Among initial isolates, pyrazinamide-monoresistant TB (PR 2.21; 95% CI 1.90–2.57) was more common, and isoniazid-resistant (PR 0.69; 95% CI 0.60–0.79) and multidrug-resistant TB (PR 0.58; 95% CI 0.39–0.85) less common among TB–HIV patients than among patients without known HIV co-infection. Pyrazinamide-monoresistant TB among TB–HIV patients increased from 2% during the pre-HAART era to 8% during the late HAART era, which was out of proportion to the increase (from 1% to 3%) among patients with TB alone (p<0.05 for difference in slopes).

#### Clinical Outcomes

Compared with the pre-HAART era, death during treatment decreased in the late HAART era among TB–HIV patients (30% vs. 14%) but not among TB patients without known HIV co-infection (6.7% vs. 6.7%; p<0.001 for difference). In multivariate analysis, older age (RR 1.3 per 10 years; 95% CI 1.2–1.4), lower CD4+ T-lymphocyte count (RR for reference value <50 cells/mm^3^ vs. >350–500 cells/mm^3^, 6.5; 95% CI 2.7–15.6), pre-HAART characteristics (RR 2.2; 95% CI 1.9–2.6), sputum smear positivity (RR 1.2; 95% CI 1.1–1.4), and female sex (RR 1.4; 95% CI 1.1–1.7) were associated with increased risk for death, whereas the heterosexual HIV risk group (relative to MSM, RR 0.6; 95% CI 0.4–0.8) was protective (Table 3). Results were similar in a sensitivity analysis excluding rather than imputing missing CD4+ T-lymphocyte data (Technical Appendix).

**Table 3:** Multivariate analysis of factors associated with deaths among HIV-infected TB patients, California, USA, 1993–2008*

Characteristic	Adjusted relative risk (95% CI)
Time period	
2001–2008	Referent
1996–2000	1.18 (0.98–1.41)
1993–1995	2.21 (1.88–2.60)
Age†	1.29 (1.22–1.36)
Female sex	1.36 (1.12–1.65)
Race/ethnicity	
White non-Hispanic	Referent
Black non-Hispanic	0.86 (0.73–1.01)
Hispanic	0.86 (0.67–1.09)
Asian/Pacific Islander	0.70 (0.11–4.52)
Foreign birth	0.65 (0.36–1.17)
HIV risk group‡	
MSM	Referent
IDU	1.02 (0.88–1.19)
Heterosexual contact	0.58 (0.43–0.78)
Unknown	1.24 (1.04–1.48)
Sputum smear-positivity	1.23 (1.07–1.40)
CD4+ T-lymphocyte count, cells/mm^3^§	
<50	6.45 (2.67–15.58)
50–99	5.57 (2.40–13.90)
100–199	3.09 (1.28–7.46)
200–349	1.47 (0.58–3.72)
350–499	Referent
>500	1.99 (0.66–6.08)
TB as AIDS-defining diagnosis¶	1.22 (1.07–1.38)

*TB, tuberculosis; MSM, men who have sex with men; IDU, injection drug user. †Per 10-year increase in age. ‡Categories are mutually exclusive; any IDU was included in the IDU category. §CD4+ T-cell counts were imputed (online Technical Appendix, wwwnc.cdc.gov/EID/article/12-1521-Techapp1.pdf). ¶TB was considered the AIDS-defining event if TB and AIDS were reported within 6 months of each other.

## Discussion

In California, a dramatic decline in TB–HIV rates coincided with the introduction of HAART and improvements in TB control. The TB–HIV intersection has evolved from one in which active TB and AIDS progressed in a marginalized, US-born population to an intersection increasingly comprising persons from areas outside the United States with elevated TB incidence who acquire HIV infection in the peri-immigration period. TB–HIV-associated death has decreased substantially but remains approximately twice that associated with TB alone.

Population declines in TB–HIV after introduction of HAART are well documented in low-income countries (*16**,**17*). Despite the resurgence of TB in the United States during 1985–1992 (*18*), overall case rates unexpectedly decreased in some metropolitan areas before the availability of HAART because of improvements in TB control (*19*); in California, specific declines in TB–HIV began before the widespread use of HAART in 1996 (Figure 1). Improvements in programmatic TB control and declines in new annual HIV infections through the 1980s resulting from HIV awareness and prevention programs might have contributed to TB–HIV declines independent of HAART availability.

As in other high-income settings (*20**,**21*) and consistent with TB and TB–HIV (*22*) trends in the United States, immigrants from low-income countries increasingly represent the face of TB–HIV in California. Increased risk for TB–HIV among newcomers from sub-Saharan Africa reflects the hyperendemic nature of TB and HIV in this region. Yet, the demographic transformation of TB–HIV in California has been characterized largely by immigrats from Mexico or Central America and, to a lesser extent Southeast Asia, regions that have concentrated rather than generalized HIV epidemics. The finding that the median time in-country for foreign-born persons with TB–HIV is 11 years has specific TB control implications. This finding suggests that HIV transmission commonly occurs during the peri- or post-immigration period (*23*), and screening for HIV and latent TB infection restricted to new immigrants will not address the large number of co-infected residents who might benefit from treatment. Furthermore, immigrants who arrive without documentation lack systematic opportunities for HIV screening. These testing gaps will translate to undetected infection and progression to AIDS unless post-entry screening for immigrants is intensified. Finally, the substantial increase in pyrazinamide monoresistance, a surrogate marker for *Mycobacterium bovis* (an organism inherently resistant to pyrazinamide) further corroborates this demographic shift because TB in the United States caused by *M. bovis* primarily occurs among Mexican immigrants exposed to unpasteurized milk products (*24*). This finding has implications for transmission, epidemiologic surveillance, and preventive interventions, as well as improvements in pyrazinamide susceptibility testing (*25*).

In the United States, new HIV infections (*26*) and TB–HIV (*22*,*27*) remain concentrated among Blacks. However, Hispanics have the highest TB–HIV rates in California, a finding corroborated by reports from municipalities along the US–Mexico border (*28*). Current rates of TB among HIV-infected Hispanics in California exceed rates in many World Health Organization–defined high prevalence TB countries but are modest compared with the high rates in southern African countries (*29*) or marginalized HIV-infected populations in pre-HAART–era United States (*30*).

The dramatic decline in TB among HIV-infected persons in California has not been accompanied by a concurrent decrease in the proportion of patients who have advanced AIDS or late diagnosis of HIV infection, and TB rates continue to far exceed background TB incidence in the state. The distribution of CD4+ T-lymphocyte counts at TB diagnosis in California, even in the late HAART era, is not dissimilar to that found in studies from sub-Saharan Africa (*31*) or Southeast Asia (*32*). The opportunities missed are costly and underscore the need for early and innovative approaches to reach immigrants who are at particular risk for both infections. Implementation research that improves understanding of barriers to HAART as provided through key existing programs, such as the AIDS Drug Assistance Program and the Ryan White HIV/AIDS Program, is needed.

Although contrary to findings in much of the published literature from regions of high TB incidence, the higher prevalence of sputum smear positivity among HIV-infected patients is consistent with that found in prior studies from the United States (*33*). The prevalence of smear positivity is a function of multiple factors that affect the denominator (e.g., completeness of case reporting, reference standard testing) and numerator (e.g., quality of laboratory services, including staff workload and smear microscopy methods), and a combination of these factors probably contribute to differences in sputum smear positivity noted in areas of high versus low TB incidence.

Deaths associated with TB–HIV have declined remarkably in California since the pre-HAART era, consistent with national trends (*7*). Yet, although postmortem diagnoses of TB decreased significantly among patients with TB and with TB–HIV, patients with TB–HIV remain more than twice as likely to die during anti-TB treatment in the late HAART era as patients without known HIV co-infection. Women with HIV infection in the United States and other high-income areas have lagged behind men with respect to declines in mortality during the HAART era (*34*).

Our study has some potential limitations. First, TB–HIV cases may be underreported because TB patients without matches in the state HIV/AIDS registry were classified as HIV-negative. Persons who did not undergo HIV testing or whose providers did not adhere to the CDC AIDS case definition at TB diagnosis might not have been reported. However, assuming HIV awareness and testing have increased over time (*35*), misclassification of TB–HIV cases has declined in a time-dependent fashion. Moreover, during 1996–2006, the sensitivity of our case match procedure for capturing AIDS cases was ≈98% (95% CI 97.3%–98.7%) (*36*), and since confidential name-based HIV reporting began in 2006, <5% of TB–HIV cases were not also reported as AIDS cases (data not shown). Second, because antiretroviral therapy was unavailable in California Department of Public Health HIV/AIDS surveillance data, risk stratification according to HAART was not possible. Third, HIV prevalence estimates are a function of multiple parameters, some of which (race-stratified HAART-coverage, HIV incubation period, and migration patterns) carry considerable uncertainty. However, our estimates were subjected to multiple sensitivity analyses and are broadly consistent with extended back-calculation procedures undertaken by CDC (*26*). Applying national HIV rates to California would yield slightly higher HIV prevalence estimates. Last, trends in TB–HIV in California may not be generalizable to the United States as a whole.

In California, declines in TB–HIV incidence and death in the HAART era have been accompanied by a demographic shift toward foreign-born persons, particularly from Mexico and Central America. The opportunities for preventing TB and AIDS among foreign-born persons are underappreciated. Documentation of HIV status for TB patients in California (66% in 2008) remains below the national average (80%) (*37*). Screening and treatment completion rates for latent TB infection also are suboptimal despite the national standard and federal benefit (through the Health Resources and Services Administration) covering this practice (*38*), Improvements are hoped for with the recent availability of shorter course latent TB infection regimens (*39*). Progress toward comprehensive TB–HIV surveillance and recent lifting of legal barriers to HIV reporting at TB diagnosis are further cause for encouragement (*40*). TB and HIV programs must collaborate to monitor the confluence and changing epidemiology to ensure early detection of HIV and TB and to avert preventable deaths.

Technical Appendix Registry cross match, nonparametric back-calculation, multiple imputation, and tables showing results of nonparametric back-calculation sensitivity analyses and of multivariate analysis of factors associated with deaths among HIV-infected TB patients.
