# Supplementary material for: Tuberculosis and HIV Co-infection, California, USA, 1993–2008
Source: Emerg Infect Dis. 2013 Mar;19(3):400–6. doi: 10.3201/eid1903.121521 (PMC3648844; doi:10.3201/eid1903.121521)
Supplement: Technical Appendix — Registry cross match, nonparametric back-calculation, multiple imputation, and tables showing results of nonparametric back-calculation sensitivity analyses and of multivariate analysis of factors associated with deaths among HIV-infected TB patients. [file 12-1521-Techapp-s1.pdf]

Article DOI: http://dx.doi.org/10.3201/eid1903.121521

Tuberculosis and HIV Co-infection, California, 1993–2008

Technical Appendix

I. Registry Cross-match

Prior to April 17, 2006, name-based reporting was restricted to confidential AIDS cases in California; after this date, confidential reporting of all verified cases of HIV infection was also required. Since the US Centers for Disease Control and Prevention (CDC) 1993 AIDS case definition, all patients with active TB and HIV infection are counted as AIDS cases (*1*). TB patients with HIV co-infection were identified through a statewide registry match with the California Office of AIDS using Registry Plus Link Plus software (*2*), a probabilistic record linkage program developed by CDC. Registry cross-match criteria included name, sex, race/ethnicity, and date and place of birth. Manual review of all matched cases was performed, with only those matches above a predetermined priority threshold considered to represent a co-infected case (*3*). Demographic, behavioral, and clinical information, including mortality, was abstracted from state surveillance forms (Report of a Verified Case of Tuberculosis and Adult HIV/AIDS Confidential Case Report). Surveillance data for both diseases have demonstrated high validity (*4*,*5*).

II. Nonparametric Back-calculation

Nonparametric back-calculation (*6*) calculates the HIV incidence rate based on AIDS diagnoses, using the incubation period distribution. During the highly active antiretroviral therapy (HAART) era, initiation of HAART prior to AIDS diagnosis by a fraction of patients leads to a slowing of HIV progression and delays AIDS diagnosis, effectively lengthening the average incubation period. Because the fraction of individuals initiating therapy is not well characterized by race over time in California, the realized distribution of the waiting time between HIV infection and AIDS diagnosis is not well known, and thus, while back-calculation may yield

informative estimates of the HIV incidence, highly precise estimates of the incidence of HIV infection cannot be obtained.

We model the number of California diagnoses in ethnicity k during year i as follows. Let I_{ik} denote the number of individuals infected during year i for ethnicity k, and let F_{ji} denote the probability of diagnosis in year j given infection in year i. In general, observed diagnosis counts in any given year depend on the following: 1) changes in the diagnosis definition as occurred, for instance, in 1992; 2) delays in reporting; and 3) migration of HIV-positive case-patients into and out of California prior to AIDS diagnosis. Note also that, in general, the quantities F_{ji} depend on the year i, due to treatment because individuals who have initiated HAART have a slowing rate of progression due to suppression of viral replication. In the absence of these features, we may use the method of nonparametric back-calculation to compute an estimate of the effective number infected during year i as follows. The effective number infected is, in general, smaller than the true number of infected, because some of those truly infected initiated HAART and were therefore less likely to become diagnosed with AIDS than individuals who had not initiated HAART; the effective number infected provides an approximate equivalent untreated HIV population size, which may be useful in computing incidence rates, but is not useful for public health planning.

To compute the nonparametric back-calculation estimate, we assume independent Poisson counts for the diagnoses in each year (effectively ignoring diagnosis delay, a reasonable assumption given the time that has elapsed since the counts we are analyzing began). Then we have the following expected number of diagnoses in year i: $\lambda_i = \sum_{\ell \le i} F_{i\ell} I_\ell$; for simplicity, we suppress the subscript k for ethnicity in what follows. Let i denote the vector of diagnoses from year $i=0$ to year N. Then, the log likelihood of a given sequence of counts y_i is $\log(L) = \log\left(\prod_{i=0}^{N} \frac{e^{-\lambda_i} \lambda_i^{y_i}}{y_i!}\right) = \sum_{i=0}^{N} \left(-\lambda_i + y_i \log(\lambda_i)\right) + c$ (where c is a term constant in the unknowns I_{i-l}). Following Bacchetti et al. (1993), we add the following penalty term. Let $\theta_i = \log(I_i)$; the penalty is $q = \sum_{i=0}^{N-2} \left(\theta_i - 2\theta_{i+1} + \theta_{i+2}\right)^2$. We then maximize $A(I) = \log(L) - kq$, where 1980 corresponds to year 0, and N=29 (for a total of 30 years of data, i.e., through 2009).

While the elegance of the nonparametric bootstrap derives in part from its relative freedom from mechanistic assumptions, our application of this method to the post-HAART period requires consideration of the change in the incubation period distribution at the population level caused by a fraction of people starting therapy. Unfortunately, the rate of testing and the rate of starting therapy at different times and for different CD4 progression stages are simply not known. We therefore derived incubation period distributions by modifying the HIV staging model developed by Longini et al (1989) (*7*) using the methods in Charlebois et al. (2011) (*8*); specifically, we derive differential equations that we will use to derive the quantities F_{ji}. To derive them, we will use a model of untreated HIV, but then assume that a certain fraction of individuals commence therapy and that their progression is slowed. For untreated individuals, the 3 stages are 1) infected but not seropositive 2), seropositive but asymptomatic, and 3) pre-AIDS symptoms and abnormal hematology (see Longini et al. 1989 for details). We used empirical estimates for the progression rates between these stages (*7*), yielding an untreated mean incubation period of approximately 10 years (consistent with other findings, e.g. Cooley et al., 1996) (*9*). For treated individuals, we assume the same stages, but with a smaller progression rate between them. We also classify individuals by status (k) (*1*): person does not know he/she are infected (*2*), person known HIV positive but untreated (*3*), person has initiated HAART but has not yet achieved full virologic suppression, and (*4*) person on HAART and on treatment with the maximum virologic suppression he/she will achieve. We let Y_{jk} denote the number of HIV-infected people in stage j and status k. We denote the testing rate in stage j at time t by $\theta_j\left(t\right)$, the rate o initiating suppressive therapy by $\phi_j\left(t\right)$, the rate of achieving full virologic suppression by ρ, the rate of discontinuation of therapy for individuals in virologic suppression by α, the progression rate among untreated individuals from stage j to stage $j+1$ by γ_j (j=1, 2, 3), and the relative progression rate in status k by s_k (s_1=1, s_2=1). Denote the incidence rate by $I(t)$ (assumed piecewise constant within each year). Mortality due to non-AIDS causes is assumed to be negligible for simplicity. The equations are then

$$\frac{dY_{jk}}{dt} = I\left(t\right)\delta_{j1}\delta_{k1} + Y_{j1}\theta_j\left(-\delta_{k1}+\delta_{k2}\right) + Y_{j2}\phi_j\left(-\delta_{k2}+\delta_{k3}\right) + \rho Y_{j3}\left(-\delta_{k3}+\delta_{k4}\right) + \\ Y_{j4}\alpha\left(-\delta_{k4}+\delta_{k2}\right) - Y_{jk}s_k\gamma_j + Y_{j-1,k}s_k\gamma_{j-1}\left(1-\delta_{j1}\right)$$

where we separate subscripted indices with a comma when the index is an expression and not a single symbol, and δ_{ij} is a Kronecker delta. Numerical integration using the R package "odesolve" allows the matrix elements F_{ji} to be computed (the initial conditions are Y_{jk}=0 for all *j* and *k*).

The numerical parameter estimates were as follows. Testing is assumed to be zero before time 5 (year 1985); treatment is zero before time 16 (year 1996). The progression parameters were derived from Longini et al (1989); γ_1=5.49 yr^{-1}, γ_2=0.228 yr^{-1}, and γ_3=0.191 yr^{-1}. Unfortunately, population-based estimates for the testing and treatment rates by race over time are unavailable; we assumed that individuals have a testing rate of 0.3 yr^{-1} prior to AIDS diagnosis (a rate which would underestimate the rate for urban men who have sex with men), a rate of cessation of antiretroviral therapy of 0.01 yr^{-1}, a rate of 4 yr^{-1} of achieving full virologic suppression (i.e., a mean duration of 3 months before achieving full suppression), and that individuals on full suppression only progress at 10% of the rate of untreated individuals (which may in fact be an overestimate). In the base case scenario, we chose the rate of treatment in stage *j*=2 to be ¼ of that in stage *j*=3, and chose the rate in stage 3 so that by 2008, 50% of individuals with HIV but no AIDS diagnosis would be on treatment. Because many of these parameters are not well characterized, we conducted sensitivity analysis.

Of particular importance are the rates of treatment and testing by race. For the base case scenario, we assumed all rates were identical; the TB rates computed from these denominators thus contain no built-in assumption of differential rates of starting therapy.

We used parametric bootstrap (Efron & Tibshirani [*10*], p. 53–5) to compute confidence intervals for rates (with 100 replications). Specifically, for the denominator data, we assumed the estimated race-stratified incubation period over time and simulated AIDS incidence data given the random incubation period distribution for each year. We then estimated the HIV incidence from these simulated data, and from the HIV incidence, we computed the prevalence as indicated above; this yields a set of changing prevalences over time reflecting the expected variability given the sample sizes. For each of these, we simulated numerator (TB case) data using an independent Poisson distribution whose mean equaled the observed number of cases for the year in question, and computed the rate given these simulated numerators and denominators, yielding confidence intervals reflecting variability in the numerator and denominator.

Confidence intervals from parametric bootstrap do not reflect variability based on modeling assumptions. We therefore conducted the following sensitivity analyses (Technical Appendix Table 1) (*1*): assuming (unrealistically) no pre-AIDS treatment at all (*2*), assuming constant treatment rates sufficient to place 90% of HIV patients on HAART prior to diagnosis by 2009 (*3*), assuming treatment rates identical to the base case for 1996–2000, but assuming treatment rates 20% larger in 2001–2005, and a further 20% larger for 2006 to the present. We also conducted sensitivity analyses using different values for the smoothing parameter; values of 0 lead to wildly unstable estimates and are unsuitable (*6*). Finally, the 1992 (and earlier) AIDS case definition changes were not explicitly modeled; sensitivity analysis in which 10% of cases diagnosed in 1992 would have occurred in 1993 and 1994, however, did not substantially change our estimates.

Optimization was conducted by the Nelder-Mead method, using 25 randomly chosen starting values to reduce the chance of convergence to a merely local maximum. All calculations were conducted by using R v. 2.13 (http://cran.r-project.org) for MacIntosh (Apple, Cupertino, CA, USA).

III. Multiple Imputation

Multiple imputation allows asymptotically unbiased estimation of missing data under an assumption of missing at random conditional on measured variables (*11*). To recover missing values for CD4+ T-lymphocyte count (1,732 of 3,904 [44.4%]) and viral load (2,756 of 3,904 [70.6%]), we constructed 10 imputation data sets (*12*) per individual using Stata 12.2 (Stata Corporation, College Station, TX, USA) '*mi*' suite of commands. CD4+ T-lymphocyte counts or HIV viral load not recorded or ascertained >6 months prior to TB diagnosis were considered "missing." Demographic (age, sex, race/ethnicity, nationality, homeless status, correctional facility) and clinical variables (year of diagnosis, time from initial HIV diagnosis to TB diagnosis, time from initial HIV diagnosis to AIDS event, whether TB was the AIDS defining event, mode of HIV transmission, vital status at time of TB diagnosis, and tuberculin skin test reading) were used to impute missing CD4+ T-lymphocyte count and viral load values. Demographic, behavioral, and clinical information, including mortality, were abstracted from state surveillance forms (Report of a Verified Case of Tuberculosis, and Adult HIV/AIDS

Confidential Case Report). TB was considered the AIDS-defining event if TB and AIDS were reported within 6 months of each other. For each individual, regression analysis was done on each imputation data set, and results were combined to ascertain estimates by using standard multiple imputation techniques (*13*). CD4+ T-lymphocyte count was square root transformed and viral load was log-transformed prior to imputation. In order to avoid working with implausible imputations in the analysis, square root transformed CD4+ T-lymphocyte counts with negative values were truncated to 1 prior to back-transformation. Multivariate associations with mortality obtained following multiple imputation and from an unimputed "complete-case" analysis are demonstrated in the main text and below, respectively (Table 3; Technical Appendix Table 2). Overall goodness of fit for the complete-case analysis was assessed by using the Hosmer-Lemeshow test.

Technical Appendix Table 1. Nonparametric back-calculation sensitivity analyses

		TB rate, 1993			TB rate, 2008		
Parameter	Value	White	Black	Hispanic	White	Black	Hispanic
Baseline	0.00	202.30	768.6	680.52	34.80	147.94	224.85
Rate of testing	1.00	202.41	762.23	678.31	34.70	149.63	225.18
Treatment rate	0	205.95	775.75	681.67	37.02	160.85	241.66
Cessation rate	0.02	202.23	776.00	679.28	35.06	150.94	230.03
Cessation rate	0.01	201.25	772.20	690.99	34.38	149.44	219.84
Relative progression	0.05	202.22	767.02	681.27	34.49	150.59	224.40
Relative progression	0.20	203.01	740.13	686.27	35.29	170.58	233.61
Treatment fraction in 2008	0.90	191.78	743.86	666.52	28.39	118.18	181.25
Increase rate, 5 y	25%	198.50	776.40	676.98	33.73	148.76	225.58
Case definition change	0	202.30	769.96	681.12	34.83	151.59	227.94

Technical Appendix Table 2. Multivariate analysis of factors associated with deaths among HIV-infected TB patients, California, 1993–2008*

Characteristic	Adjusted relative risk (95% CI)
Time period	
2001–2008	Referent
1996–2000	1.07 (0.83–1.36)
1993–1995	2.13 (1.71–2.66)
Age†	1.29 (1.22–1.36)
Sex, F	1.33 (1.03–1.73)
Race/ethnicity	
White, non-Hispanic	Referent
Black, non-Hispanic	0.85 (0.66–1.09)
Hispanic	1.03 (0.77–1.36)
Asian/Pacific Islander	0.93 (0.58–1.47)
Foreign birth	0.68 (0.53–0.88)
HIV risk group‡	
MSM	Referent
IDU	1.08 (0.87–1.34)
Heterosexual contact	0.58 (0.39–0.86)
Unknown	1.35 (1.08–1.70)
Sputum smear positivity	1.23 (1.02–1.49)
CD4+ T-lymphocyte count, cells/mm^3	
<50	5.21 (2.14–12.64)
50-99	3.69 (1.50–9.07)
100–199	2.92 (1.20–7.14)
200–349	2.25 (0.88–5.80)
350–499	Referent
>500	1.59 (0.50–5.06)

Characteristic	Adjusted relative risk (95% CI)
TB as AIDS-defining diagnosis§	0.94 (0.78–1.13)

*Complete-case analysis (CD4+ T-cell counts not imputed. TB, tuberculosis; MSM, men who have sex with men; IDU, injection drug use.
†Per 10 year increase in age.
‡Categories are mutually exclusive; any IDU was included in the IDU category.
§TB was considered the AIDS-defining event if TB and AIDS were reported within 6 months of each other.

References

1. Centers for Disease Control and Prevention. 1993 Revised classification system for HIV infection and expanded surveillance case definition for AIDS among adolescents and adults. MMWR Recomm Rep. 1992;41(RR-17):1–9. PubMed

2. Plus Version L. 2.10 probabilistic record linkage software. Atlanta: Centers for Disease Control and Prevention; 2006 [cited 2012 Dec 11]. http://www.cdc.gov/cancer/npcr/tools/registryplus/lp.htm

3. Qiang X, Westenhouse JL, Schultz AF, Nonoyama A, Elms W, Wu N, et al. Matching AIDS and tuberculosis registry data to identify AIDS/tuberculosis comorbidity cases in California [cited 2012 Dec 11]. Health Informatics J. 2011;17:41–50. http://jhi.sagepub.com/content/17/1/41.

4. Sprinson JE, Lawton ES, Porco TC, Flood JM, Westenhouse JL. Assessing the validity of tuberculosis surveillance data in California. BMC Public Health. 2006;6:217. PubMed http://dx.doi.org/10.1186/1471-2458-6-217

5. Klevens RM, Fleming PL, Li J, Gaines CG, Gallagher K, Schwarcz S, et al. The completeness, validity, and timeliness of AIDS surveillance data. Ann Epidemiol. 2001;11:443–9. PubMed http://dx.doi.org/10.1016/S1047-2797(01)00256-3

6. Bacchetti P, Segal MR, Jewell NP. Backcalculation of HIV infection rates. Stat Sci. 1993;8:82–119. http://dx.doi.org/10.1214/ss/1177010994

7. Longini IM Jr, Clark WS, Byers RH, Ward JW, Darrow WW, Lemp GF, et al. Statistical analysis of the stages of HIV infection using a Markov model. Stat Med. 1989;8:831–43. PubMed http://dx.doi.org/10.1002/sim.4780080708

8. Charlebois ED, Das M, Porco TC, Havlir DV. The effect of expanded antiretroviral treatment strategies on the HIV epidemic among men who have sex with men in San Francisco. Clin Infect Dis. 2011;52:1046–9. PubMed http://dx.doi.org/10.1093/cid/cir085

9. Cooley PC, Myers LE, Hamill DN. A meta-analysis of estimates of the AIDS incubation distribution. Eur J Epidemiol. 1996;12:229–35. PubMed http://dx.doi.org/10.1007/BF00145410

10. Efron BTR. An introduction to the bootstrap. New York: Chapman and Hall; 1993.

11. Klebanoff MA, Cole SR. Use of multiple imputation in the epidemiologic literature. Am J Epidemiol. 2008;168:355–7. PubMed http://dx.doi.org/10.1093/aje/kwn071

12. Harel O, Zhou XH. Multiple imputation: review of theory, implementation and software. Stat Med. 2007;26:3057–77. PubMed http://dx.doi.org/10.1002/sim.2787

13. Rubin DB. Multiple imputation for nonresponse in surveys. New York: Wiley; 1987.
